# Hydration and Properties of Magnesium Potassium Phosphate Cement Modified by Granulated Blast-Furnace Slag: Influence of Fineness

**DOI:** 10.3390/ma15030918

**Published:** 2022-01-25

**Authors:** Kuisheng Liu, Shanliang Ma, Zengqi Zhang, Fanghui Han

**Affiliations:** 1 Beijing Urban Construction Group Co., Ltd., Beijing 100084, China; liuks@mail.bucg.com; 2 School of Metallurgical and Ecological Engineering, University of Science and Technology Beijing, Beijing 100083, China; g20208295@xs.ustb.edu.cn; 3 School of Civil and Resources Engineering, University of Science and Technology Beijing, Beijing 100083, China; hanyang-1120@163.com

**Keywords:** magnesium potassium phosphate cement, slag, fineness, workability, compressive strength

## Abstract

Magnesium potassium phosphate cement (MKPC) is an excellent rapid repair material for concrete, and many mineral admixtures have been applied to promote its performance. This study focuses on the quantitative characterization of the physical and chemical contributions of granulated blast-furnace slag with various finenesses to the performance development of MKPC. It was found that the addition of slag could increase the setting time, which is mainly due to the dilution of cement. Fine slag tends to decrease the fluidity of MKPC mortar. The physical contributions of ordinary and ultrafine slag to the early performance of MKPC mortar are 23% and 30%, while the chemical contributions are only 6%~10%. At late ages, the physical contribution is less than 10% and the chemical contribution of slag is even slightly negative. The addition of slag is beneficial to the compact packing of MKPC, which is the main reason for the physical contribution. Slag could react in the MKPC system, and increasing the fineness significantly promotes the reaction kinetics.

## 1. Introduction

Magnesium potassium phosphate cement (MKPC) has been widely used as a rapid repair material for concrete structures due to its excellent bond strength [1,2,3], high early performance [4] and high volume stability [5]. The acid-base reaction between dead-burned magnesia (MgO) and potassium dihydrogen phosphate (KH_2_PO_4_) is the driving force of the microstructure development and property growth of MKPC [5,6]. Due to the high reactivity and rapid reaction rate, MKPC presents fast setting and hardening properties even at an extremely low temperature [5,7]. In addition, MKPC presents a high encapsulation capacity for heavy metals [8,9], and thus, it is also used as a storage material for treatment of some solid wastes. The main reaction product of MKPC is K-struvite, and the main factors controlling the performance of MKPC are the water/binder ratio (W/B) and magnesia-to-phosphate molar ratio (M/P) [10,11,12,13,14].

Generally, MKPC is a kind of Mg-based chemically bonded phosphate ceramic. The reaction mechanism of Ca-based, Al-based, Fe-based and Zn-based chemically bonded phosphate ceramics [15] provided valuable guidance for the modification of MKPC with various mineral admixtures. Mineral admixtures with high CaO content (e.g., wollastonite) have been proven to be beneficial to the properties of MKPC due to the formation of calcium phosphate [16,17]. The precipitation of aluminum phosphate (AlPO_4_) gel due to the reaction of active aluminum in MKPC also led to a compact cement matrix [1]. Some solid wastes with high FeO content were also found to be beneficial to the early property development of MKPC with a constant M/P ratio. One of the main chemical compounds of most solid waste is SiO_2_, and it has been reported that the active silica in some solid waste could react in MKPC, forming M-S-H and improving the performance (but the effectiveness is limited due to the low reactivity of dead-burned magnesia in M-S-H-forming reactions) [16]. Therefore, fly ash [4,18,19], granulated blast-furnace slag [20,21,22], silica fume [23,24], metakaolin [1,25], wollastonite [16,17] and some other mineral admixtures containing alkali aluminosilicate components have been used to modify the MKPC. In addition to the possible chemical reactions, the physical effect of the mineral admixtures could also play an essential role, optimizing the microstructure and further enhancing the performance of MKPC [26].

Slag has been widely used as a supplementary cementitious material in Portland cement [27,28,29,30,31] or geopolymer concrete [32,33]. Ultrafine grinding has been proven to be effective in improving the reactivity of slag [34]. The hydration characterization and properties of ultrafine slag blended cement have been widely reported [35,36,37]. However, research on MKPCs modified by ultrafine slag is quite limited.

In this study, the effect of the fineness of slag on the properties of blended MKPC was quantitatively characterized. The contributions of slag to the mechanical properties due to the chemical reaction and physical effect were calculated. The influence of slag fineness on the workability of fresh MKPC mortar was also investigated. By particle packing simulation, isothermal calorimetry testing, BSE/EDS mapping and aqueous solution property measurement, the physical and chemical mechanisms of slag on the performance development of MKPC were analyzed.

## 2. Materials and Methods

### 2.1. Raw Materials

**Figure 1:**
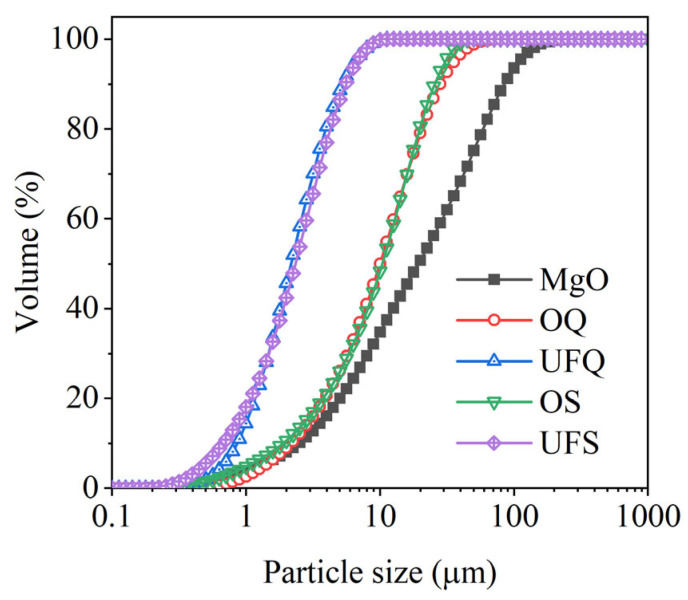
Particle size distributions of raw materials. OQ: ordinary quartz; UFQ: ultrafine quartz; OS: ordinary slag; UFS: ultrafine slag.

Dead-burned magnesia (MgO) with a purity of 92% and KH_2_PO_4_ of industrial grade are the main composition of MKPC. Granulated blast-furnace slags with various levels of fineness were chosen as mineral admixtures. Inert quartz with a similar particle size distribution of slag was selected as a reference. The chemical compositions of these binders were determined by X-ray fluorescence (XRF) analysis and are presented in Table 1. The particle size distributions of magnesia, slag and quartz are given in Figure 1. It is obvious that the fineness of ordinary and ultrafine quartz are similar to those of ordinary and ultrafine slag, respectively.

### 2.2. Methods

#### 2.2.1. Mixture Proportions

Three systems of composite MKPC were investigated in this study, i.e., mortar with a W/B of 0.16, paste with a W/B of 0.5 and suspension with a W/B of 5.0. The mixture proportions are presented in Table 2. Mortar, paste and suspension are named as ‘M-’, ‘P-’ and ‘S-’, respectively. The dosage of quartz or slag is denoted with a suffix. For example, sample M-20%OQ denotes the mortar prepared by replacing 20% MgO with ordinary quartz. The magnesia-to-phosphate molar ratio (M/P) in composite MKPC was fixed at 6.0. Borax was used as the retarder, and the mass ratio of retarder/MgO was fixed at 5% during the preparation of the mortar.

#### 2.2.2. Test Methods

The composite MKPC mortars were prepared with dimensions of 40 × 40 × 40 mm^3^ according to the mixture proportions in Table 2. The mortar samples were cured under uniform conditions (25 °C and 60% RH). After 1, 3, 7, 28 and 90 d, the compressive strength of the mortar was measured at a loading rate of 2.4 MPa/s.

The setting time of the composite MKPC pastes with a W/B of 0.16 was determined by the Vicat needle test according to ASTM C191. Only the final setting time was determined since it is quite close to the initial setting time of MKPC pastes. The fluidity of the fresh MKPC pastes were examined by the mini-cone slump method.

The hydration heat of the MKPC pastes with a water/binder ratio of 0.5 was measured by isothermal conduction calorimetry (TA instrument, New Castle, DE, USA) at 25 °C.

Scanning electron microscope (SEM, HITACHI SU8220, Tokyo, Japan) was used to observe the microstructure of samples P-40%OS. The different phases could be clearly distinguished according to the gray level distributions. Energy-dispersive X-ray spectroscopy analyses (EDS, spot and mapping) were used to characterize the phase compositions. Before observation, the sample was immersed in epoxy resin and then polished by an automatic polish-grinding machine (EcoMet 30, BUEHLER, Lake Bluff, IL, USA).

The pH value of the suspension was monitored in situ by a SANXIN pH meter connected to a PC, and data were automatically collected and recorded every 10 s.

## 3. Results and Discussion

### 3.1. Properties of the Fresh MKPC Paste

**Figure 2:**
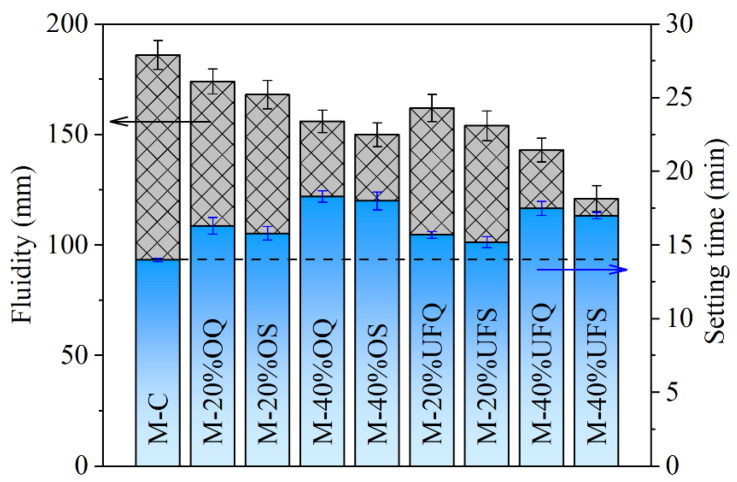
Fluidity and setting time of the fresh MKPC mortar.

The fluidity and setting time of the fresh MKPC paste are presented in Figure 2. The addition of ordinary inert quartz leads to 16.42% and 30.71% increases in the setting time of MKPC pastes at replacement ratios of 20% and 40%, respectively. The setting is caused by the rapid reaction of cement, generating the hydration products and forming a mechanically irreversible network of cement grains [38]. The increasing effect of mineral admixture on the setting time of MKPC paste is contributed to the dilution effect on the cement (decreasing the cement content and increasing the real water/cement ratio [39]), and thus, the increasing effect is much more obvious with a high replacement ratio. The increasing effect of quartz on the setting time of fresh MKPC paste becomes less obvious with a smaller particle size. This phenomenon is supposed to be caused by the accelerating effect of finer particles on the hydration of MKPC, which is widely acknowledged in Portland cement systems (e.g., the seeding or filler effect) [40,41]. Compared with that of quartz, the increasing effect of slag on the setting time is smaller due to the extra chemical reaction. The 20% ordinary and ultrafine slags result in only 12.85% and 8.57% increases in setting time, respectively, which are significantly smaller than those of the system containing quartz with similar fineness. The physical and chemical effects of slag also lead to a decrease in the fluidity of fresh MKPC paste. The physical effects of 40% ordinary and ultrafine quartz powder (assuming fully physical effect, without chemical effect) lead to 16.13% and 23.11% decreases in the fluidity, respectively. On that basis, the chemical reactions of ordinary and ultrafine slag (assuming the same physical effect as the quartz powder because of their particle sizes; the rest is attributed to chemical effect) further result in 6.98% and 15.59% decreases in fluidity.

### 3.2. Compressive Strength of MKPC Mortar

**Figure 3:**
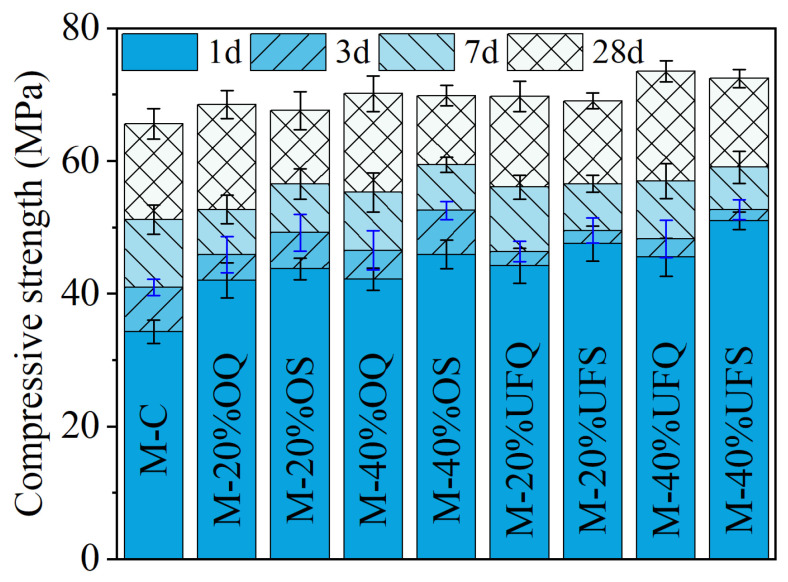
Compressive strengths of the MKPC mortar.

Figure 3 shows the compressive strength of the MKPC mortar. It could be found that the addition of quartz increased the compressive strength of the MKPC mortar, especially at 1 day. Forty percent ordinary and ultrafine quartz resulted in extra 8 MPa and 11.2 MPa growth of the 1 day’s compressive strength. Particle size is the dominant property that significantly affects the physical filler effect of minerals. It was found that the physical effect of slag is approximately identical to that of quartz with a similar fineness in Portland cement system [42,43,44]. Therefore, the contribution of inert quartz to the development of mechanical properties could be roughly regarded as a representation of the physical filler effect of slag since they have similar particle size distributions (see Figure 1). Additionally, the 1-day compressive strengths of samples M-40%OS (ordinary slag) and M-40%UFS (ultrafine slag) are 3.71 MPa and 5.46 MPa larger than those of samples M-40%OQ and M-40%UFQ, respectively. This gap contributes to the chemical reaction of slag, which promotes the precipitation of extra reaction products. The extra compressive strength of the MKPC mortar containing slag decreases at late ages. The 28-day compressive strength of the mortar containing slag is even lower than that containing inert quartz. This means that the addition of slag mainly promotes the growth of the early mechanical properties of MKPC mortar due to both physical and chemical effects. The contribution of slag to the mechanical properties of MKPC mortar at late ages is limited, and the chemical reaction of slag even presents a slight negative effect on the late-age strength development. This negative effect could be attributed to the incorporation method of slag: addition to the system instead of replacing MgO, while maintaining constant W/B. In this way, the reaction of slag competes phosphate with the reaction of MgO. Since the MgO-to-K-struvite reaction represents an extremely large volume expansion [14], much larger than the reaction of slag, this competition declines the overall pore-filling efficiency of the chemical reactions; and, thus, leads to lower (than expected) compressive strength at later ages. In the case of quartz, no chemical competition occurs, so all phosphate is dedicated to K-struvite formation, maintaining high pore-filling efficiency and strength.

**Figure 4:**
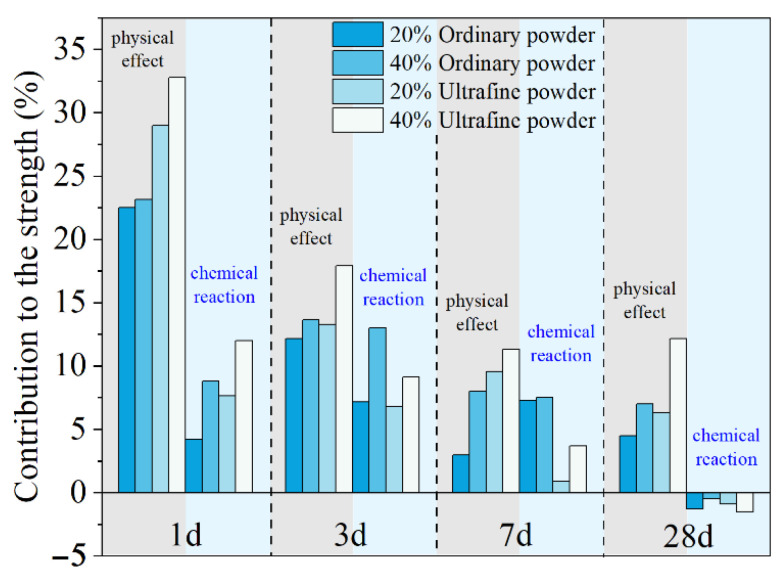
Quantitative contributions of the physical and chemical effects on the mechanical properties of MKPC mortar.

**Figure 5:**
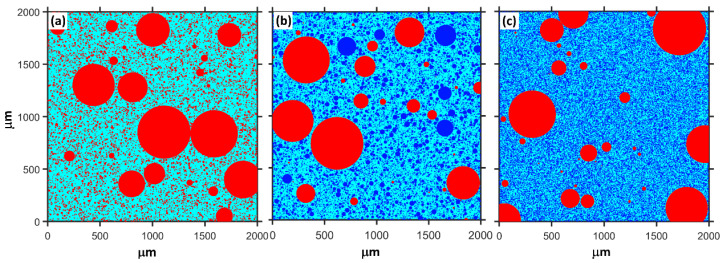
Schematic diagram of the random distribution of MgO and mineral admixtures in a box with a length of 2000 microns, M/P = 6, W/B = 0.16. Red: MgO; Blue: mineral admixture. (**a**) only MgO (**b**) with 40% ordinary powder and (**c**) with 40% ultrafine powder.

To quantitatively characterize the physical and chemical effects of slag on the strength development of MKPC mortar, a contribution ratio was calculated as follows:

(1) $$R_{physical}=\left(C_{Q}-C_{Ref}\right)/C_{Ref}\times 100\%$$

(2) $$R_{chemical}=\left(C_{S}-C_{Q}\right)/C_{Q}\times 100\%$$

where $C_{Ref}$, $C_{Q}$ and $C_{S}$ are the compressive strength of plain MKPC mortar and MKPC mortar containing quartz and slag, respectively. The quantitatively calculated results are presented in Figure 4. The physical effect of ordinary powder results in a 22–23% increase in the compressive strength after 1 day. However, this contribution of ultrafine powder is 29–33%. As shown in Figure 1, MgO is mainly composed of particles larger than 50 μm. The addition of fine or ultrafine powders is beneficial to the compact packing of the MKPC mortar [45,46,47] (see Figure 5). Therefore, the physical effect of ordinary and ultrafine slags is supposed to be contributing to the fine particle size, which makes the paste achieve the maximum packing density. However, this physical filler effect becomes less obvious at late ages due to the dilution of the MKPC, and thus, the physical contribution of fine powders to the strength is less than 10% at 28 days.

The contributions of ordinary and ultrafine slag to the early strength due to their chemical reaction are approximately 6% and 10%, respectively. The higher reactivity of the ultrafine slag brings a larger strength development at 1 day. However, at 7 days, the chemical reaction of ordinary slag still leads to approximately 7% extra strength development, while the positive contribution of ultrafine slag is only 3%. At 28 days, the contributions of both ordinary and ultrafine slags to the strength of MKPC mortar are slightly negative.

### 3.3. Properties of the MKPC Paste

**Figure 6:**
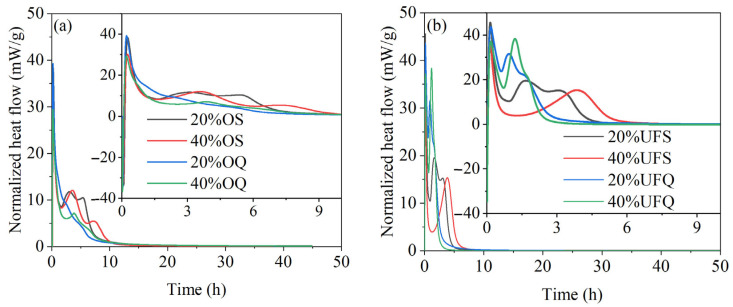
Exothermic rates of MKPC containing quartz or slag. (**a**) addition of ordinary powder; (**b**) addition of ultrafine powder.

The exothermic rates of MKPC with quartz and slag are given in Figure 6. It could be found in Figure 6 that the second major exothermic rate peaks of samples containing ultrafine quartz are much more obvious than those of the samples containing ordinary quartz. The exothermic rate of the sample with ultrafine quartz at the second major hydration peak exceeds 30 mW/g, while that of the sample with ordinary quartz is smaller than 10 mW/g. This result could be explained by the high filler effect (nucleation effect) of ultrafine powders. The chemical reaction of ordinary slag could be clearly found in the exothermic rate curve. The exothermic rate of the sample with ordinary slag reaches 13 mW/g at the second major hydration peak, which is 50% higher than that of the sample with ordinary quartz. However, the chemical reaction of ultrafine slag significantly decreases the second major exothermic rate from 30 mW/g to 20 mW/g. Meanwhile, the second major hydration peak shifted to the right with the reaction of ultrafine slag. It is supposed that the rapid reaction of ultrafine slag consumes part of the KH_2_PO_4_ and thus partially delays the reaction of MgO at the second major reaction peak. Despite this, the exothermic rate of the sample with ultrafine slag is still approximately twice as high as that of the sample with ordinary slag. The reaction rate of the sample with ultrafine slag is much higher than that of the sample with ordinary slag, and thus, the second major exothermic peak ends 4 h earlier. It could be concluded that the chemical reaction of ordinary slag contributes more than the physical effect of ordinary slag on the hydration heat. However, in the case of ultrafine slag, the contribution of the physical effect to the early hydration heat is higher than that of the chemical reaction. The increase in slag fineness could enhance both the physical and chemical effects, but the enhancement of the physical effect is more significant.

**Figure 7:**
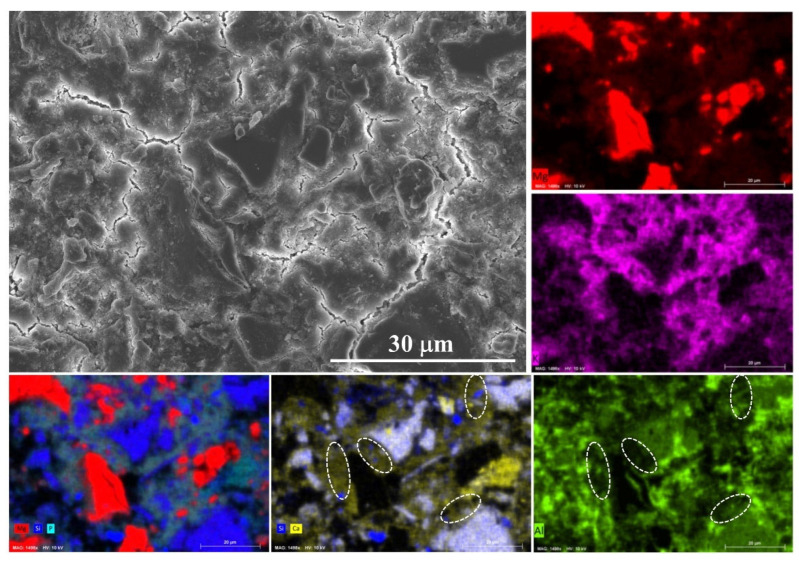
Typical SEM image and EDS mapping result of sample P-40%OS at 28 days.

The SEM image and the related EDS mapping results are presented in Figure 7. The particle size of MgO is much larger than that of the ultrafine slag, which would seriously affect the BSE/EDS mapping analysis results. Therefore, the reaction mechanism is investigated by the sample with 40% ordinary slag at 28 days. As shown in the EDS mappings, the reaction products are composed of Mg, K, P, Si, Al and Ca. It means that slag could react in MKPC system. The most interesting finding is that the content of Ca is higher than that of Si in reaction products, likely indicating that the dissolution of calcium in slag is easier and could diffuse over greater distances. The alumina polishing paste was used during the polishing process of the sample, and thus the Al mapping result may be influenced by the residual polishing paste. However, it could still be found in the unaffected areas (marked by dash line) that there are few aluminum elements in the reaction products. This finding provides evidence for the CaO-phosphate reaction hypothesis regarding slag-MKPC interaction mechanisms [20,21].

**Figure 8:**
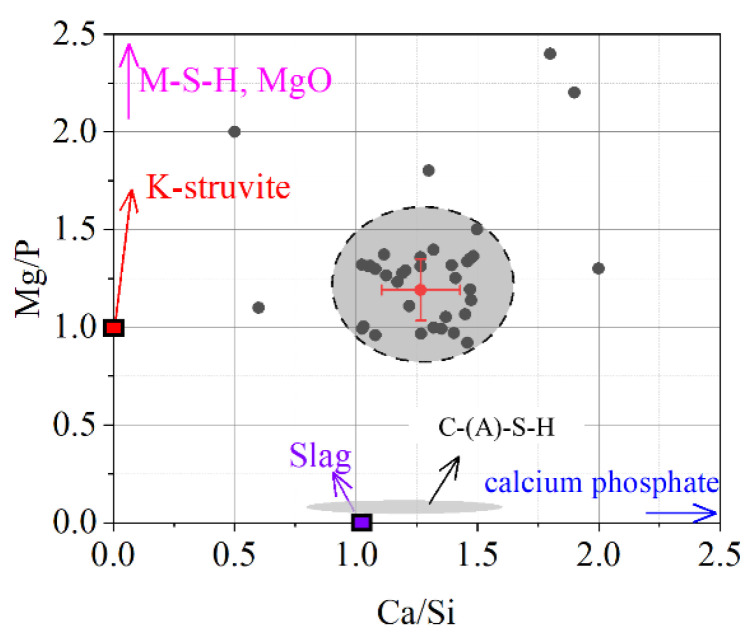
Chemical composition of the reaction product.

The chemical composition of the reaction products in ordinary slag blended MKPC paste was measured by EDS spot scanning analysis. By considering the Mg and Si mapping results, the EDS spots selected in Figure 7 could be strictly distributed in the products and away from the unreacted magnesium oxide and slag. The EDS results of the reaction products are given in Figure 8. Most of the EDS points fall in the circular area with Mg/P molar ratios from 0.9 to 1.5 and Ca/Si molar ratios from 1.0 to 1.4. The average molar ratios of Mg/P and Ca/Si are approximately 1.19 and 1.27, respectively. It is suggested that the reaction products of slag-blended MKPC could be mixtures of K-struvite, M-S-H (in small chance), C-(A)-S-H and calcium phosphate. The formation of amorphous product and calcium phosphate were also found by Peng and Chen [48] and Tan et al. [49].

### 3.4. Properties of the MKPC Suspensions

**Figure 9:**
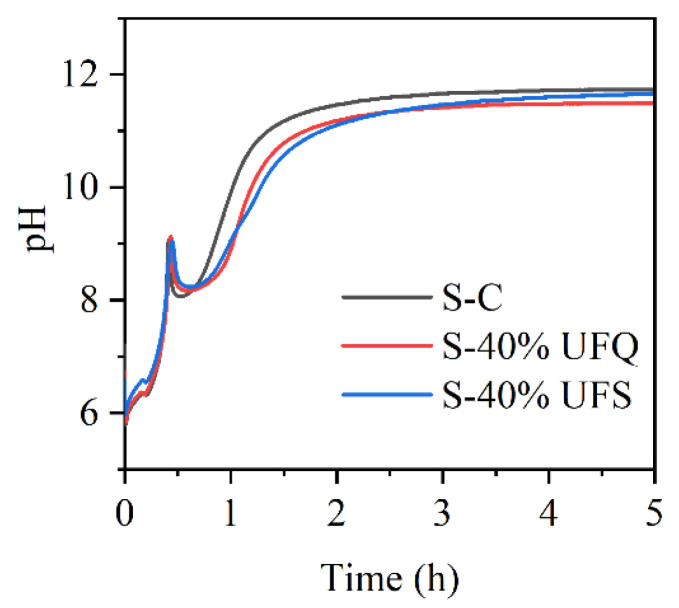
pH curves of the suspensions with ultrafine quartz or slag.

**Figure 10:**
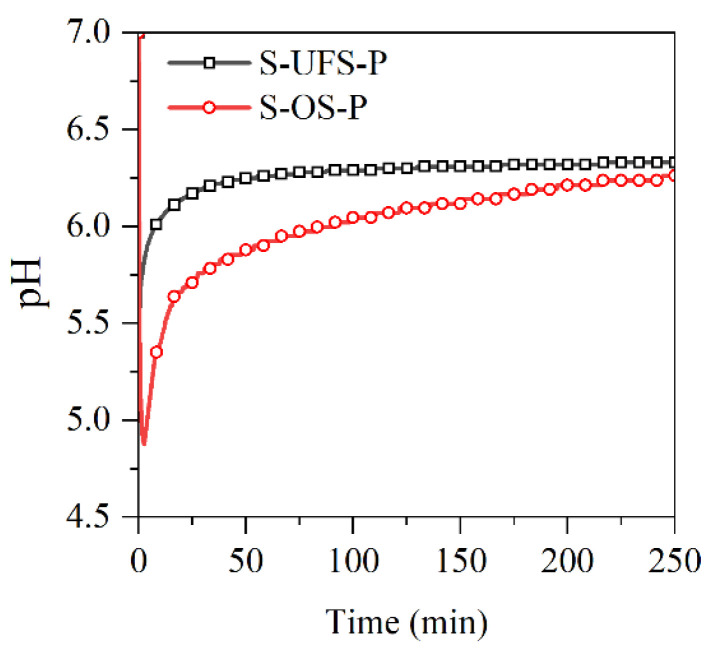
pH curves of the slag/KH_2_PO_4_ suspensions.

The pH values of the MKPC suspensions with ultrafine quartz or slag were monitored in situ and are presented in Figure 9. The pH values in all suspensions drop quickly due to the rapid dissolution of KH_2_PO_4_. The resultant acidic environment facilitates dissolution of MgO (and slag components), which results in gradual increasing of the pH value. In this process, gel-like reaction products could be formed, which thicken to form K-struvite precipitates at a pH of ~9, leading to pH drop. When the K-struvite formation reactions are stabilized, the slow dissolution of MgO starts to dominate the pH evolution process. The pH values of the suspensions containing quartz or slag are lower than those of the plain MKPC system after 1 h due to the dilution of MgO. However, the final alkalinity of the suspension with slag is almost identical to that of the plain MKPC. To investigate the influence of the fineness of slag on the reaction kinetics with KH_2_PO_4_, the pH values of the suspensions prepared by KH_2_PO_4_ and ordinary/ultrafine slag were measured in situ and are presented in Figure 10. The minimum pH of the suspension was approximately 4.8, which appeared at approximately 5 min. The ultrafine slag reacted rapidly in the acidic solution, and thus, the pH value of the suspension increased to a constant (approximately 6.3) within half an hour. The ordinary slag also reacted in the KH_2_PO_4_ suspension, and the final pH value of the suspension was almost equal to that of the suspension with ultrafine slag. However, the reaction rate of ordinary slag is relatively slow, and it takes 5 h to reach constant alkalinity.

## 4. Conclusions

The aim of the present research was to examine the influences of the fineness of slag on the hydration and properties of MKPC. The contributions of ordinary or ultrafine slag to the workability of fresh MKPC, the mechanical properties and the hydration heat of MKPC due to the physical filler effect and chemical reaction were quantitatively calculated. The following conclusions could be drawn.

The addition of quartz or slag could increase the setting time and decrease the flow ability of MKPC paste. Ordinary quartz or slag (40%) led to an approximately 29% increase in setting time and an 18% decrease in fluidity. However, the 40% ultrafine quartz or slag only resulted in an approximately 23% increase in setting time. The decrease effect of ultrafine powder on the fluidity reaches 23–35%. Increasing the fineness of slag weakens both the increasing effect in setting time and the decreasing effect in fluidity.

The addition of slag could enhance the early mechanical performance of MKPC mortar due to both the physical filler effect and chemical reaction. The increase in fineness could further promote the positive contribution of slag to the early strength development of MKPC. The physical contribution of ordinary slag to the early strength of MKPC mortar is approximately 23%, and it could exceed 30% with increasing fineness. The chemical contributions of ordinary and ultrafine slag to the early strength of MKPC mortar are approximately 6% and 10%, respectively. At late ages, the physical contribution is less than 10%, and the chemical contribution is even slightly negative.

The particle packing simulation proved that the fine slag particles are beneficial to compact packing and enhance the performance of the MKPC mortar. Isothermal calorimetry, BSE/EDS mapping and aqueous solution properties proved that slag could react in the MKPC system and that increasing the fineness could obviously promote the reaction of slag.

## Figures and Tables

**Table 1:** Chemical compositions of raw materials (*wt*/%).

Compound	Fe_2_O_3_	SiO_2_	Al_2_O_3_	SO_3_	CaO	MgO	P_2_O_5_	K_2_O	Na_2_O	Others
Magnesia	1.00	2.41	0.41	-	3.58	91.67	0.53	0.02	-	0.38
KH_2_PO_4_	0.01	0.08	0.03	-	0.13	0.39	52.58	45.11	1.38	0.29
slag	0.92	35.75	14.26	1.95	35.14	9.66	-	-	0.86	1.46
Quartz	0.01	99.90	0.04	-	0.01	0.01	-	-	0.01	0.02

**Table 2:** Mixture proportions.

Sample	M/P Ratio ^a^	w/b Ratio ^b^	w/c Ratio ^b^	MgO	KH_2_PO_4_	OS/UFS	OQ/UFQ	b/s Ratio ^c^
MKPC mortars								
M-C	6	0.16	0.16	1.78	1	-	-	1:1
M-20%OQ/UFQ	6	0.16	0.186	1.78	1	-	0.445	1:1
M-20%OS/UFS	6	0.16	0.186	1.78	1	0.445	-	1:1
M-40%OQ/UFQ	6	0.16	0.195	1.78	1	-	0.89	1:1
M-40%OS/UFS	6	0.16	0.195	1.78	1	0.89	-	1:1
MKPC pastes								
P-C	6	0.5	0.5	1.78	1	-	-	-
P-20%OQ/OQ	6	0.5	0.58	1.78	1	-	0.445	
P-20%OS/OS	6	0.5	0.58	1.78	1	0.445	-	
P-40%OQ/UFQ	6	0.5	0.66	1.78	1	-	0.89	-
P-40%OS/UFS	6	0.5	0.66	1.78	1	0.89	-	-
Suspensions								
S-C	6	5	-	1.78	1	-	-	-
S-40%UFQ/UFS	6	5	-	1.78	1	0.89	-	-
S-OS/UFS-P	6	5	-	-	1	1	-	-

^a^ fixed molar ratio. ^b^ w/b represents the mass ratio between water and binders (binders include magnesia, KH_2_PO_4_, slag and quartz); w/c represents the mass ratio between water and cement (cement includes magnesia and KH_2_PO_4_). ^c^ binder to sand mass ratio.

## Data Availability

The data presented in this study are available on request from the corresponding author.
